# Breakage-Reunion Domain of *Streptococcus pneumoniae* Topoisomerase IV: Crystal Structure of a Gram-Positive Quinolone Target

**DOI:** 10.1371/journal.pone.0000301

**Published:** 2007-03-21

**Authors:** Ivan Laponogov, Dennis A. Veselkov, Maninder K. Sohi, Xiao-Su Pan, Aniruddha Achari, Cheng Yang, Joseph D. Ferrara, L. Mark Fisher, Mark R. Sanderson

**Affiliations:** 1 Randall Division of Cell and Molecular Biophysics, School of Biomedical and Health Sciences, King's College London, London, United Kindgom; 2 Molecular Genetics Group, Molecular and Metabolic Signalling Centre, Division of Basic Medical Sciences, St. George's, University of London, London, United Kindgom; 3 Rigaku Americas Corporation, The Woodlands, Texas, United States of America; Institute of Molecular and Cell Biology, Singapore

## Abstract

The 2.7 Å crystal structure of the 55-kDa N-terminal breakage-reunion domain of topoisomerase (topo) IV subunit A (ParC) from *Streptococcus pneumoniae*, the first for the quinolone targets from a gram-positive bacterium, has been solved and reveals a ‘closed’ dimer similar in fold to *Escherichia coli* DNA gyrase subunit A (GyrA), but distinct from the ‘open’ gate structure of *Escherichia coli* ParC. Unlike GyrA whose DNA binding groove is largely positively charged, the DNA binding site of ParC exhibits a distinct pattern of alternating positively and negatively charged regions coincident with the predicted positions of the grooves and phosphate backbone of DNA. Based on the ParC structure, a new induced-fit model for sequence-specific recognition of the gate (G) segment by ParC has been proposed. These features may account for the unique DNA recognition and quinolone targeting properties of pneumococcal type II topoisomerases compared to their gram-negative counterparts.

## Introduction

DNA topoisomerases are enzymes of vital cellular importance due to their ability to alter DNA topology. Replication of duplex DNA or recombination between DNA duplexes may cause the formation of DNA supercoils, knots and catenanes. The major role of topoisomerases is to control DNA topology by transient breakage of DNA strands. This activity is achieved through the formation of covalent enzyme-DNA complexes linked via phosphotyrosine bonds. Type I topoisomerases act on one strand of DNA whereas type II enzymes pass a DNA duplex through a double-strand DNA break, resulting in changes of DNA linking number in units of 1 and 2, respectively [1]–[3].

Type II topoisomerases comprise a family of structurally and evolutionarily conserved enzymes common to all organisms [4] and have been implicated in a variety of intracellular processes including DNA replication, transcription and chromosome segregation [5]. Because of their importance to the cell life cycle, these enzymes have been a subject of intense drug development studies and were identified as targets for a large number of natural toxins, antimicrobial agents and anti-tumour therapeutics [6]–[8].

Based on sequence and structural criteria, type II topoisomerases are divided into two subclasses. The most common subclass, type IIA, includes the bacterial enzymes topo IV and gyrase, and eukaryotic topo II. Type IIB topos are structurally and biochemically distinct, and comprise a single family member, topo VI [2], [9]–[11]. All members of the type IIA subclass exhibit significant sequence and mechanistic similarity.

Bacterial genomes usually encode two type IIA enzymes, topo IV and DNA gyrase, each composed of two subunits (ParC and ParE in topo IV, GyrA and GyrB in gyrase) assembled into a functional heterotetramer *i.e.* C_2_E_2 _and A_2_B_2_, respectively [6]. Both enzymes act by passing a duplex DNA segment through a transient double-stranded DNA break in another helical region. In the currently accepted model [9], the open enzyme clamp binds a DNA segment termed the ‘gate’ or G-segment. The N-terminal ATPase domains of the ParE (GyrB) subunits dimerize upon ATP binding capturing the DNA duplex to be transported (T-segment). The T-segment is then passed through a transient break in the G-segment (opened by the N-terminal ParC (GyrA) domains), the DNA is resealed and the T-segment released through a protein gate prior to resetting of the enzyme to the open clamp form.

Although topo IV and gyrase share a high degree of similarity, their cellular functions are different. Gyrase controls DNA supercoiling and relieves topological stress arising from the translocation of transcription and replication complexes along DNA. Gyrase is unique in its ability to introduce negative supercoiling into the DNA. By contrast, topo IV is a decatenating enzyme that resolves interlinked daughter chromosomes following DNA replication. The functional differences between gyrase and topo IV are attributed to the non-specific binding of the C-terminal domain of the GyrA subunit that wraps DNA presenting a neighbouring T-segment for passage through the G-gate. Removal of this domain converts gyrase into an enzyme with topo IV-like activities, i.e. ATP-dependent DNA relaxation and decatenation [12].

In addition to their unusual mechanisms, topo IV and gyrase are also targets of clinically important quinolone drugs. Quinolones exert their bactericidal effects by interfering with DNA breakage-reunion by topo IV and gyrase leading to double-stranded DNA breaks at specific sites. Recent studies have uncovered fundamental differences in quinolone action in gram-positive vis-à-vis gram-negative bacteria. For example, though gyrase is the universal target of quinolones in gram-negative bacteria, topo IV is the primary (or dual) target of many quinolones in gram-positive pathogens such as *Streptococcus pneumoniae*, a major cause of pneumonia and other serious infections [13], [14]. Second, pneumococcal topo IV is more sensitive than gyrase to quinolone inhibition in vitro, the inverse of that seen in *E. coli* [15]. Third, quinolones trap *S. pneumoniae* topo IV (and gyrase) at DNA sites (G-segments) that are distinct in sequence from those observed for *E. coli* topo IV and gyrase [15]. These unique features emphasize the need to analyze the structure of *S. pneumoniae* topo IV and how it binds quinolones and DNA. Progress in these areas will be important in understanding ParC-mediated resistance to quinolones and in developing dual targeting drugs that minimize the emergence of resistance [13], [16], [17].

## Results and Discussion

**Figure 1:**
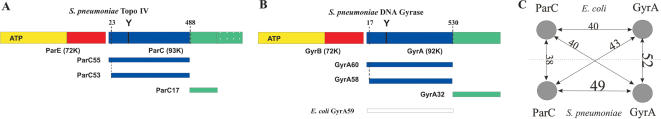
Comparison of type II topoisomerases from *S. pneumoniae* and *E. coli*. Schematic domain organization of (A) *S. pneumoniae* topoisomerase IV, (B) *S. pneumoniae* gyrase (with the proteolytic fragments generated by trypsin digestion superimposed) and (C) graphical representation of the sequence similarity scores for 500-residue N-terminal fragments of ParC (P72525) and GyrA (P72524) from *S. pneumoniae* and equivalent fragments of ParC (P0AFI2) and GyrA (P0AES4) from *E. coli* calculated using ClustalW [49].

**Figure 2:**
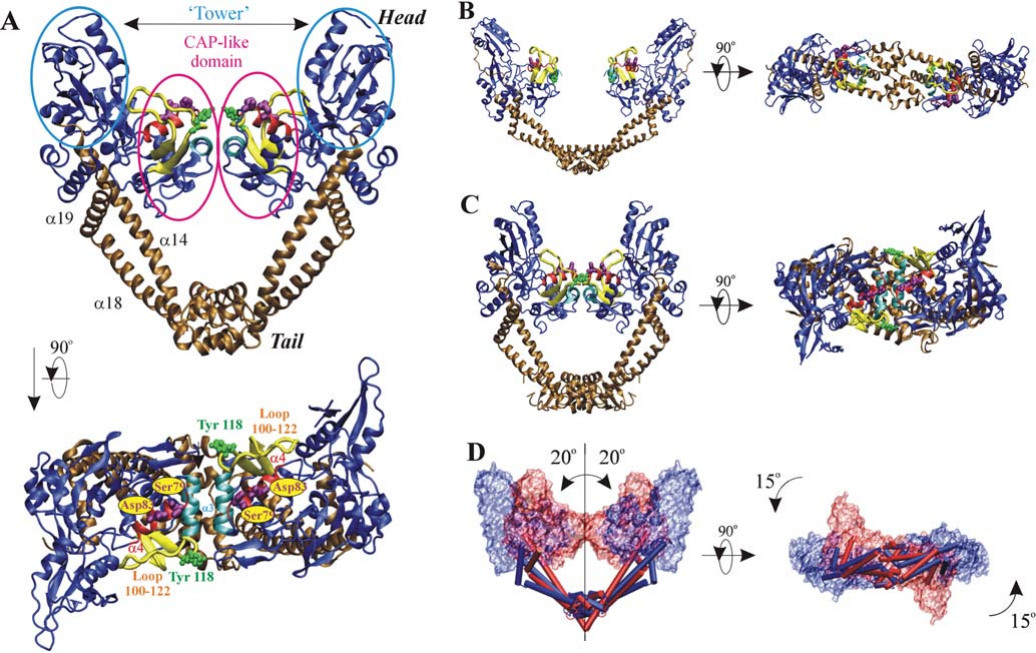
Structure of the ParC55 dimer and illustration of structurally related *E. coli* ParC and GyrA proteins. (A) Orthogonal views of the ParC55 biological dimer from *S. pneumoniae*. (B) Structure of the N-terminal region of *E. coli* ParC (18) (1ZVU) equivalent to ParC55 fragment. (C) Structure of the N-terminal fragment (GyrA59) of *E. coli* GyrA [19] (1AB4). In (A), (B) and (C) the ‘towers’ and the CAP-like domains are shown in ice blue; the ‘tails’ along with adjacent helices α14, α18 and α19 are in ochre; the helix α4 in red; the helix α3 in cyan and the 100-122 loop in yellow. The active-site tyrosines are shown in green. Residues Ser 79 and Asp 83 responsible for drug-resistance upon mutation are in purple. (D) Schematic conversion of ParC55 from ‘closed’ (red) to ‘open’ (blue) conformation on the basis of the *E. coli* ParC structure. Panels were rendered using VMD [50] and Pov-Ray.

Digestion with trypsin revealed that pneumococcal ParC is organized into a 53-kDa N-terminal domain (ParC53, residues 23-487) and a compact 17-kDa C-terminal region (Figure 1A). ParC53 was enzymatically inactive whereas a recombinant ParC55 fragment (residues 1-490), when complemented with topo IV ParE, reconstituted quinolone-promoted DNA cleavage (data not shown). The DNA cleavage specificity of the ParC55-ParE complex was identical to that of pneumococcal topo IV and exhibited the same unique base recognition preferences of −4G/+8C, −2A/+6T and+1G/+4C (relative to the DNA scission site between nucleotide −1 and+1). These determinants are very different from those of *E. coli* topo IV (preferences −1(A/G) and+1(T/A)) and *E. coli* gyrase (a degenerate 20-bp consensus) [15]. Sequence alignment with the N-terminal domains of *E. coli* GyrA and ParC whose crystal structures have been solved [18], [19] shows greater similarity of *S. pneumoniae* ParC55 with *E. coli* GyrA and *S. pneumoniae* GyrA (forms a GyrA58 (residues 17–529) tryptic fragment via GyrA60, Figure 1B) than with *E. coli* ParC (Figure 1C). However, these comparisons do not explain the distinctive enzyme preferences in G-segment recognition. To gain insight into the mechanism of *S. pneumoniae* topo IV and its interaction with DNA and quinolones, the structure of the ParC55 fragment has been solved (Figure 2A) and the model was refined to R = 22.30% (R-free = 27.55%) at 2.7 Å resolution using wild-type ParC55 data (twinning fraction = 0.323, twinning operator = (k,-h,l)). The crystallization of ParC55, the quinolone target from gram-positive bacteria, and X-ray analysis were first reported by our group in Acta Cryst. (2005) A61, C176.

### Crystal structure of ParC55: a closed dimer with a distinctively charged DNA binding groove

ParC55 forms a biological dimer in a ‘closed’ conformation in this crystal form generating a ring-like structure with outer dimensions of 100×100×50 Å and with a central hole approximately 30 Å in diameter, which is wide enough to pass a duplex DNA (Figure 2A). This is in contrast to the ParC structure from *E. coli* which adopts an ‘open’ dimer conformation [18] (Figure 2B), but similar to the closed dimer of GyrA59 from *E. coli* [19] (Figure 2C). Each monomer of ParC55 contains two distinct regions labeled ‘head’ and ‘tail’ and exhibits an overall structural fold similar to the one observed for other topoisomerases [18]–[20]. The N-terminal proximal head contains the CAP-like (catabolite gene activator protein [21]–[23]) DNA-binding domain and the ‘tower’. The CAP-like domain is composed of a helix-turn-helix structural motif (helices α3 and α4) typical for DNA-binding proteins, with the active site Arg 117 and Tyr 118 on the neighboring ‘100-122’ loop (Figure 2A). The residues Ser 79 and Asp 83, which upon mutation give rise to quinolone-resistant pneumococci, are located primarily within the helix α4 in close proximity to the active-site tyrosine and are oriented towards the predicted position of the bound DNA. The ‘tower’ is positioned on the top of the CAP-like domain and has largely α-helical and β-sheet structural organization. High B-factor values and poor definition of several loops indicate a high level of flexibility in this region. In the closed conformation, the ParC55 monomers form a tight contact between the α3 helices with a mean center-to-center distance of 10 Å resulting in the protein-protein interface with a buried surface area of 706 Å^2^. This is less than in the case of *E. coli* GyrA where the monomers form a protein-protein interface with a buried surface area of 1,380 Å^2^. The ‘recognition’ α4 helices are further apart from each other by 5 Å in the case of the ParC55 dimer compared to the *E. coli* GyrA dimer.

The ‘tail’ has a primarily α-helical core composition and forms the so-called ‘primary’ dimer interface with a total buried surface area of 2,001 Å^2^. It is very similar to equivalent domains from *E. coli* GyrA and *E. coli* ParC in terms of the overall core structure, though its outer appearance is different from the ‘tail’ present in *E. coli* GyrA due to a lack of extra surface loops (Figure 2A, C). Two long helices (α14 and α18) emanate from the ‘tail’ domain and join it to the ‘head’ domain (α14 directly and α18 through a flexible loop and helix α19, respectively).

**Figure 3:**
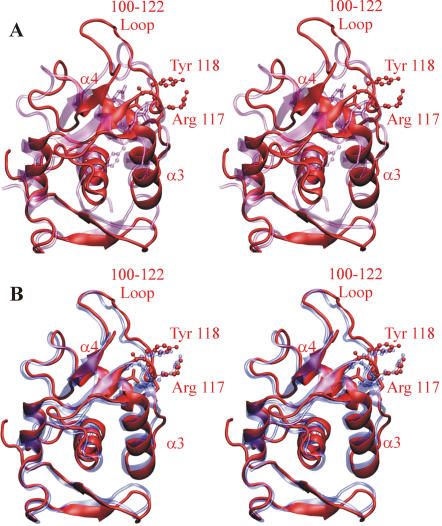
Structural comparison between active sites of topoisomerases from gram-positive and gram-negative bacteria. (A) Stereo view of the superposition of the active site of ParC from *S. pneumoniae* (red) and ParC from *E. coli* (violet). (B) Stereo view of the superposition of the active sites of ParC from *S. pneumoniae* (red) and GyrA from *E. coli* (blue). The active-site tyrosines and arginines are represented in CPK mode. Sites responsible for drug-resistance when mutated are represented in Licorice mode. Panels were rendered using VMD [50] and Pov-Ray.

Superposition of the ‘closed’ *S. pneumoniae* ParC55 and ‘open’ *E. coli* ParC dimers reveals several significant structural differences potentially relevant to topo IV catalysis (Figure 2D). The process of changing conformation from the ‘closed’ form to an ‘open’ one will involve movement of the ‘heads’ away from each other, increasing the average distance between them by approximately 30 Å and rotating them by 15 degrees relative to the tail protein dimer interface and 20 degrees relative to the vertical axis of the dimer. This movement is primarily based on changes in relative orientation of the long helices (α14, α18 and α19) which join the ‘heads’ to the ‘tails’ (Figure 2D) [18]. Interestingly, in addition to several positional changes in the α-helices and β-sheets forming the CAP-like domains, perhaps the most significant difference between *S. pneumoniae* and *E. coli* ParC structures is in the fold of the 100-122 loop containing active-site Arg 117 and Tyr 118 residues (Figure 2B and Figure 3A). In the ‘closed’ *S. pneumoniae* ParC structure, the loop is positioned vertically and stands on the side of the DNA-binding groove orienting the active-site tyrosine towards the expected position of the DNA backbone and exposing the active-site helices (α3 and α4) to the predicted protein-DNA interface. By contrast, in the *E. coli* ParC, this loop forms two additional turns one of which flips the active-site tyrosine upside-down and moves it away from the dimer interface and surface of the DNA-binding groove. In addition, the loop bends into the DNA-binding groove and covers the active-site helices α3 and α4. It seems possible that the open *E. coli* ParC structure corresponds to the ‘DNA-free’ form of topoisomerase IV and that the evident flexibility of the 100-122 loop is necessary to allow DNA binding to the closed enzyme conformation.

**Figure 4:**
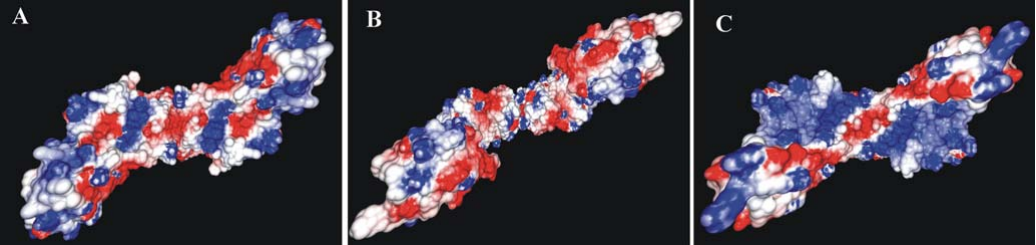
Electrostatic surface potential calculation. GRASP2 [51], [52] electrostatic surface potentials within the DNA-binding grooves calculated for equivalent N-terminal fragments of: (A) *S. pneumoniae* ParC (ParC55), (B) *E. coli* ParC, (C) *E. coli* GyrA. The *E. coli* ParC structure was manually brought into the ‘closed’ conformation, but the experimentally determined fold of the active site was preserved. Negatively charged surfaces are in red and positively charged surfaces are in blue.

*S. pneumoniae* ParC55 folds into a similar closed conformation to that of *E. coli* GyrA (Figure 3B) However, interestingly, the DNA binding site formed by the pneumococcal ParC55 dimer differs markedly in its electrostatic charge distribution from both the *E. coli* ParC and GyrA dimers (Figure 4). In particular, unlike *E. coli* GyrA which has uniform positive charge within the DNA-binding groove (except for the DNA scission active site and sides of the neighboring ‘towers’), the DNA-binding groove of *S. pneumoniae* ParC exhibits a pattern of alternating areas of positive and negative charge (compare Figures 4A and 4C). These areas correspond to the predicted positions of the backbone phosphates and of the major/minor grooves of the bound DNA strand (see below). To determine whether these distinctive charge distributions were retained across bacterial species, we modeled a number of GyrA and ParC sequences on to the *E. coli* GyrA structure (Figure S1). Modeling of *S. pneumoniae* ParC recapitulated the experimental charge distribution seen in ParC55. *S. pneumoniae* GyrA conformed almost exactly to the *E. coli* GyrA pattern. The ParC and GyrA proteins of *Staphylococcus aureus* behaved like their pneumococcal counterparts. There were also clear differences between the cognate ParC and GyrA proteins of the Gram-negative species *Haemophilus influenzae* and less so for *Neisseria gonorrhoeae*. It is possible that charge distribution differences relate to functional differences in DNA recognition and processing by the two enzymes. Thus, *E. coli* GyrA uses its C-terminal domain to bend DNA up to 180° presenting a T-segment *in cis* for passage through the G-segment and thereby introducing negative supercoils [24]–[28]. Unlike gyrase which has a 120–150-bp DNA footprint [29]–[31], topo IV lacks negative supercoiling activity and generally binds a short region of G-segment DNA (∼30 bp) [32]. The C-terminal DNA binding domain of ParC is used to guide intermolecular T segment translocation facilitating the decatenation of linked DNA molecules [18], [32]. It remains to be determined whether differences in DNA substrate recognition and processing by topo IV and gyrase are partially facilitated by differences in charge distribution in the N-terminal DNA binding site of ParC/GyrA.

### Induced-fit model for sequence-specific DNA binding and cleavage by ParC

**Figure 5:**
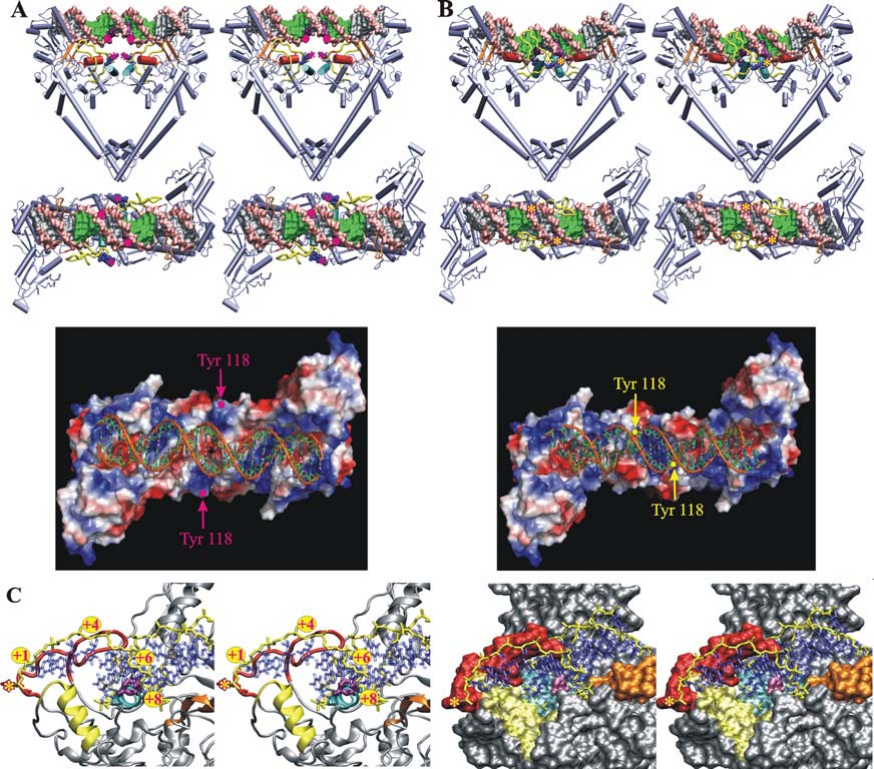
DNA binding, recognition and cleavage by *S. pneumoniae* ParC. Initial (A) and final (B) stages of DNA binding and recognition are modeled and shown as orthogonal stereo representations of the protein-DNA complex (top) together with the electrostatic surface potential of the DNA binding groove (bottom). The ParC dimer is shown in cartoon mode; the helix α4 is in red, the helix α3 in cyan, the 100-122 loop in yellow and Gly 166 - Pro 179 domain in orange. The active-site tyrosines are shown in blue color using CPK mode and indicated by asterisks (magenta for unbound and yellow for bound to the DNA states). The target 5′-end phosphates of the DNA molecule are indicated by circles in magenta. The DNA molecule is represented as a molecular surface model; the backbone is in pink, the cleavage points in purple, the base pairs involved in sequence specific recognition in green and non-specific base pairs in silver. (C) Stereo view of the active site of the modelled protein-DNA complex of *S. pneumoniae* ParC after cleavage and separation of the DNA fragments in cartoon (left) and surface (right) representation. Topo IV cleaves DNA with a 4-base stagger and the active site tyrosines are covalently attached to the 5′-ends of the DNA. Ser 79 and Asp 83 which upon mutation lead to quinolone resistance in bacteria are shown in purple, the helix α4 is in cyan, the helix α3 in yellow, the 100-122 loop with active site tyrosine in red and Gly 166-Pro 179 domain in orange. DNA molecule backbone is shown in yellow and side chains are in blue. The position of the covalent bond between the active-site tyrosine and the target 5′-end DNA backbone phosphate is indicated by a yellow asterisk in a magenta circle. Panels were rendered using VMD [50], Pov-Ray and PyMOL [53].

In the ParC55 dimer, the catalytic Tyr 118 residues are 30 Å apart and are not therefore optimally placed for attack of phosphodiester bonds in the G-segment required for covalent ParC-DNA linkage and DNA breakage. This situation also applies to the *E. coli* GyrA dimer for which it was proposed that the G-segment is bound in a distorted (perhaps single-stranded) form placing phosphodiester bonds close enough for tyrosine attack [19]. Based on the ParC55 structure and the apparent conformational flexibility of the 100-122 loop (Figure 3), we suggest a possible alternative to the previously proposed DNA “bubble” mechanism [15]. In the new model, the catalytic tyrosines are moved to attack DNA as a natural consequence of G-segment recognition and binding by the α3/α4 helices and by the 100-122 loop. Based on a knowledge of the charge distribution for the DNA-binding groove of *S. pneumoniae* ParC, differences in folding the 100-122 loop between *S. pneumoniae* ParC and *E. coli* ParC and the known DNA sequence preferences for *S. pneumoniae* ParC, we have modeled the protein-DNA binding, DNA sequence recognition and cleavage process using a 24-mer duplex corresponding to the strong E binding site of topo IV [15] (Figure 5 and Movie S1). First, approximately two turns of DNA helix are captured by the middle regions of the protein heads positioning the phosphate backbone of the DNA molecule against the positively charged grooves on the surface of the CAP-like domains and neighboring towers, with the major groove of the DNA against negatively charged middle regions of the CAP-like domains. The two β-sheets with a joining loop comprising amino acids Gly 166 to Pro 179 form a tight contact with the major groove of the DNA (Figure 5A). In the next stage, the helix α4 enters the minor groove of the DNA molecule constituting the first step in DNA sequence recognition. The possibility of the helix-turn-helix motif binding the minor groove has previously been reported for human DNA repair protein *O* ^6^-alkylguanine-DNA alkyltransferase (AGT) [33]. Alternative binding of the helix α4 to either of the neighboring areas of the major groove of the DNA seems less attractive as it would require a strong distortion of the active site which would physically push the active site tyrosines away from the target backbone phosphates.

Driven by the process of the helix α4 binding to the minor groove of the DNA, the helix α3 reorients itself moving into the major groove of the DNA bringing the 100-122 loop closer to the same groove. Then, the negatively charged 100-122 loop folds onto and binds to the DNA major groove providing the second step of DNA sequence recognition. As a direct consequence of DNA recognition and binding by the 100-122 loop, the active site tyrosine is moved close to the phosphate backbone of the DNA allowing DNA cleavage to occur (Figure 5B). The process of protein-DNA binding most likely involves slight opening of the protein dimer interface (by approximately 9 Å) and some bending of the DNA molecule towards the active site of the protein (an estimated radius of DNA curvature of about 70 Å).

The model attractively accommodates key features of topo IV action. First, unlike the proposal for DNA-binding to *E. coli* GyrA, the model requires minimal distortion of protein and DNA molecules [19]. Electrostatic potential calculated for the final stage of the DNA binding process shows that the DNA backbone is properly positioned against the positively charged regions of the protein with the DNA major/minor grooves positioned against the negatively charged regions of the DNA-binding groove along the entire DNA molecule (Figure 5B). Second, it explains the mechanism of selective DNA cleavage as arising from the DNA sequence being ‘sampled’ both from the major groove (mainly by the 100-122 loop and to some extent by the helix α3) and from the minor groove (by the helix α4). Nucleotides+4, +6 and+8 (+1, −2 and −4 on the opposite strand) are placed close to the 100-122 loop and the helices α3/α4 (Figure 5C) consistent with the strong DNA cleavage determinants observed at these positions for pneumococcal topo IV [15]. Involvement of the 100-122 loop in DNA sequence recognition may also help explain the divergence of the DNA sequence preferences between ParC and GyrA from different organisms despite very high conservation of the residues within helices α3 and α4 which are involved in protein-DNA contact formation (Figure S2).

Finally, the ParC55 structure and modeling of DNA recognition are relevant to quinolone action. At the final stages of DNA binding and cleavage (Figure 5C), the quinolone resistance sites (Ser 79 and Asp 83, which are located within the helix α4 bound to the minor groove of the DNA molecule) will be positioned next to the 3′-end of the cleaved DNA strand with Ser 79 being closest to the point of cleavage (Figure 5C). It is known that quinolones bind strongly to topoisomerase-DNA complexes stabilizing the cleaved form, but very weakly to the protein or duplex DNA alone [34], [35]. Moreover, quinolones preferentially bind single-stranded DNA [34]. These features indicate that the 3′-end of the cleaved DNA (-1 position) is a potential site for quinolone binding due to likely distortion of the double-stranded DNA at the cleavage point, as supported by clerocidin labeling experiments [36]. Sequence homology (Figure 1C) and similarity in the disposition of mutational hotspots in ParC and GyrA (Figure 3B) suggest that selective quinolone targeting of ParC in *S. pneumoniae* and GyrA in *E. coli* arises from structural conservation of high affinity drug binding sites. The *S. pneumoniae* ParC55 structure presented here provides a basis for approaching key aspects of DNA recognition and quinolone action in gram-positive bacteria.

## Materials and Methods

### Trypsin digestion of *S. pneumoniae* ParC and GyrA

ParC protein (10 µg) was incubated for 1 hour at 37°C with bovine pancreatic trypsin (0.25 to 4 µg) in reaction buffer containing 50 mM Tris-HCl, pH 7.5, 55 mM KCl, 2 mM MgCl_2_, 5 mM DTT and 10% glycerol (total volume of 20 µl). Reactions were stopped by addition of SDS to a final concentration of 1% followed by boiling for 5 minutes. Protein products were separated in a NuPAGE^TM^ 4–12% Bis-Tris gradient gel run at 100 Volts for 2.5 hours and stained with Coomassie blue. Proteins were electroblotted onto polyvinylidene fluoride membrane and their N-terminal sequences were determined using the Edman procedure developed by Dr. Arthur Moir, University of Sheffield.

For tryptic digestion of *S. pneumoniae* GyrA protein, a time course was performed. The reaction mixture contained 90 µg of GyrA and 2.25 µg of trypsin in the same digestion buffer used for ParC (total volume of 150 µl). At 0, 0.5, 1, 2, 5, 10 and 30 min, a 20 µl aliquot was removed and the reaction was stopped. Proteins were analysed by electrophoresis and sequenced as described for ParC. Recombinant ParC, ParE, GyrA and GyrB were prepared as described previously [8].

### Expression of *S. pneumoniae* ParC55

A 1.4-kb fragment of the *parC* gene of *S. pneumoniae* strain 7785 was amplified by PCR using forward primer N6894, 5′-TGGGCTTTGTATCATATGTCTAAC (NdeI site overlapping the initiation ATG codon is underlined) and reverse primer VPC4 (5′-TAGCTGTATCAATCTCGAGT-GCTTTCGCAG, *parC* nucleotide position 1482 to 1452, engineered XhoI site underlined). The NdeI-XhoI digested PCR product was ligated into pET29a, yielding an expression plasmid that in *E. coli* inducibly expressed the 55.5 kDa N-terminal ParC domain (ParC55). The protein constitutes residues 1-490 of *S. pneumoniae* ParC bearing a C-terminal hexahistidine tag and a single conserved amino acid substitution (I489L) necessary for plasmid construction. Selenomethionine-substituted ParC55 was prepared by induction in *E. coli* strain B834(DE3)pLysS (Novagen). To produce ParC55 Cys426, the *parC* Arg426 codon of the expression plasmid was altered using the Quikchange site-directed mutagenesis kit (Stratagene). Mutant proteins were overexpressed in *E. coli* and purified by Ni^2+^-NTA chromatography using the same conditions as for wild-type ParC55.

### Crystallization, data collection, structure solution and refinement

The first crystallization of ParC55, the quinolone target from gram-positive bacteria, and preliminary X-ray data analysis were reported by our group (in Acta Cryst. (2005) A61, C176).

The purified ParC55 protein was dialyzed against 20 mM Tris-HCl, pH 7.0, 200 mM NaCl, 10% glycerol, 1 mM β-mercaptoethanol, 0.05% NaN_3_ then concentrated to 3-4 mg/ml using PEG 35,000 at 4°C. ParC55 was crystallized by hanging drop vapor diffusion in 24-well Limbro plates using 0.5 ml reservoir volumes. Hanging drops were formed by mixing equal volumes of protein and crystallization solutions. Crystals grew at ambient temperature within 3 to 10 days and diffracted to 2.7 Å. The best diffracting wild-type ParC55 crystals were grown from 100 mM Tris-HCl, pH 5.5, 200 mM NaCl, 1 mM β-mercaptoethanol, 0.05% NaN_3_ and 10% of 1:1 ethanol-isopropanol as precipitant. Cys426 mutant was crystallized from 100 mM Tris-HCl, pH 6.5, 200 mM NaCl, 1 mM β-mercaptoethanol, 0.05% NaN_3_ using 4–8% PEG 400 as a precipitant and 1 mM of hexamine cobalt chloride as an additive.

The crystals were soaked for 3 to 5 seconds in cryoprotectant solutions composed of either the crystallization solutions plus 25% glycerol or 30% MPD, 150 mM NaCl, 0.05% NaN_3_, 20 mM Tris-HCl, pH 7.0 and immediately frozen in a nitrogen cryostream.

Data sets were collected at ESRF (Grenoble, France), SRS (Daresbury Laboratory, UK) and at Rigaku Americas Corporation (The Woodlands, Texas, USA), processed and integrated by HKL2000 [37] (ParC Cys426) and XDS [38] (wild-type ParC55) software. The cell dimensions for the best diffracting ParC crystal were: *a* = 136.92 Å, *b* = 137.85 Å, *c* = 326.02 Å, α = β = γ = 90.0°. Relatively high R_cryst_ (7–20% for different crystals) together with high mosaicity (0.8°–1.4°) and peculiarities of the diffraction patterns indicated that the crystal lattice was twinned. The space group was established to be I222 with non-crystallographic symmetry operators mimicking the symmetry of the I4 space group. In addition, most of the crystals were twinned with the twinning operator (k,-h,l) equivalent to one of the symmetry operators within I4. Data sets were tested for twinning using the UCLA Merohedral Crystal Twinning Server [39] and CNS [40]. The twinning fraction varied from 0.32 to 0.43 for different data sets. The only data set which did not show signs of twinning was the one collected on the Cys426 mutant using cobalt hexamine chloride as an additive. The resolution of this data was 3.25 Å and the model could not be refined to an R factor of lower than 30%, which is probably due to an unaccounted contribution from cobalt.

Several attempts to derive experimental *ab initio* phases from the Se-Met and heavy atom derivatives of ParC were unsuccessful most likely due to the high complexity of the problem, i.e. high values of the twinning fraction and the twinning operator being close to the NCS operator. The structure was solved by Molecular Replacement. The best two data sets in terms of resolution (wild-type ParC55) and completeness (Cys426 mutant ParC55) were used for the structure solution and refinement (Table 1).

**Table 1:** Summary of the crystallographic analysis

Data set	Wild type ParC55	ParC Cys426
Radiation source	ESRF Grenoble, Beam Line BM 30A	Rigaku MicroMax-007HF generator fitted with a chromium anode and a R-AXIS IV++detector
Wavelength (Å)	0.91694	2.29090
Resolution (Å)	2.67	3.25
Space group	I222	I222
Cell dimensions		
*a, b, c* (Å)	136.92,137.85, 326.02	136.39, 137.64, 328.52
α, β, γ (°)	90, 90, 90	90, 90, 90
Completeness (%)	97.2	96.0
Redundancy	5.4	8.2
R_sym_(%)*	9.3 (21.2)	7.3 (34.4)
No of sulfur sites (per monomer)	16	17
Resolution (Å)	2.7	3.25
R/R_free_(%)**	22.30/27.55	31.14/34.46
r.m.s. bond lengths (Å)	r.m.s. bond angles	
0.013	1.72	

* R_sym_ = ΣΣ*_j_*|<*I*>*-I_j_|/Σ*<*I*>.

** R/R_free_ = Σ||*F*_obs_|-|*F*_calc_||/|*F*_obs_|, where the working and free R-factors are calculated using the working and free reflection sets, respectively. The free reflections were held aside throughout refinement.

The initial model was built using 3D-JIGSAW [41]–[43]. Due to a higher sequence similarity, the GyrA from *E. coli* was picked automatically as a prototype [19]. The rotational search performed in CNS using the *E. coli* GyrA-based model gave the same rotational solutions for different data sets and the results were confirmed by AMoRe (CCP4) [44], [45]. The initial translational searches in I222 space group were not successful either in CNS or in AMoRe. The data set collected on Cys426 mutant with chromium wavelength (λ = 2.29 Å) was integrated in P1 space group and used for a thorough translational search to overcome this problem. The monomers were introduced into the translational search in P1 space group one-by-one in different orientations derived from the previous cross-rotational search and I222 space group symmetry operators. One sequential search solution resulted in formation of a reasonable biological dimer and its results were transferred back into the I222 space group giving a half of the asymmetric unit content. The asymmetric unit was completed in I222 space group using NCS-related dimer introduced into the translational search. Finally, the asymmetric unit was established to be composed of two biological dimers related to each other by an approximately 90 degree rotation around the *c* axis.

The structure was refined in CNS through several cycles of simulated annealing/positional/B-factor refinement using strict NCS operators in the initial cycles and restrained NCS operators in the final ones. Manual backbone and side chain refitting were performed using Coot [46]. The model was refined to R = 22.30% (R-free = 27.55%) at 2.7 Å resolution using wild-type ParC55 data (twinning fraction = 0.323).

**Figure 6:**
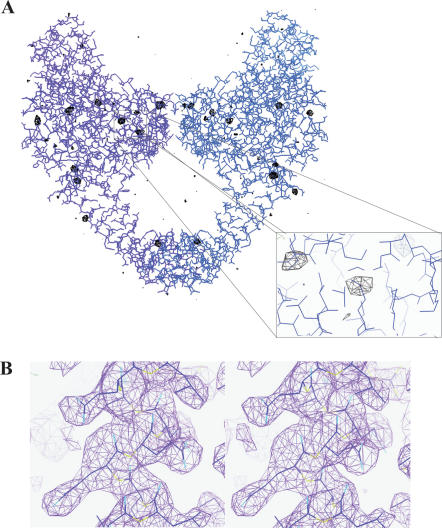
Electron density maps confirming the molecular replacement solution. (A) Superposition of the anomalous difference Fourier map calculated using Cys426 data collected on the chromium edge contoured at 3.5σ with the final refined model of ParC55. Panels were rendered using Coot [46]. (B) Stereo view of a region of the 2F_obs_-F_calc_ electron density map from wild-type ParC55 data contoured at 1.5σ with the final refined model superimposed.

The model was cross-checked using the anomalous data collected on the chromium edge for the Cys426 mutant. An anomalous difference Fourier map was generated in CCP4 [44] using the ParC Cys426 data set collected at the chromium wavelength and phases from the refined wild-type ParC55 model. The map showed clear anomalous peaks around sulfur atoms of corresponding methionines as well as cysteine 426, confirming the molecular replacement solution (Figure 6A).

Close inspection of the 2F_obs_-F_calc_ electron density maps built using both wild-type ParC55 and Cys426 mutant data sets have shown a very good coverage for most of the backbone and side-chains (ure 6B). The overall B-factors of the structure are relatively high with the average value of 67.42 Å^2^ and are similar to those shown for GyrA from *E. coli*, which indicates a high level of flexibility of the molecules [19]. The terminal residues (1 to 27 and 483 to 496) as well as the residues in flexible regions:

Chain A: 172, 283–285, 291–295, 301–304, 408;

Chain B: 172, 299, 408;

Chain C: 306, 408–409;

Chain D: 110, 171–174, 253, 282–284, 316–318, 408–409

were omitted from the final structure because no convincing electron density was observed for them.

The final Ramachandran plot indicated that most of the residues are in the favored regions: 83.2% most favored, 13.8% allowed, 2.3% generously allowed, 0.7% disallowed according to PROCHECK [47], [48].

Data deposition: The atomic coordinates and structure factors have been deposited with the Protein Data Bank, www.rcsb.org (PDB ID code 2NOV).

## Supporting Information

Figure S1 Electrostatic surface potential modeling. GRASP2 [51], [52] electrostatic surface potentials within the DNA-binding grooves calculated for DNA-binding domains of topoisomerases IIA from different organisms using models generated by 3D-JIGSAW [41]–[43] on the base of the known structure of *E. coli* GyrA (1AB4).
(3.64 MB PDF)

Figure S2 Modeling of the DNA sequence recognition by topoisomerase IV. (A) Amino acid sequence alignment for the regions of GyrA and ParC from *E. coli* and *S. pneumoniae* comprising helices α3 and α4 and the 100-122 loop. The residues within the α-helices which are likely to interact directly with the incoming DNA helix are indicated by color (red for α3 and blue for α4) and asterisks. Active site tyrosines and arginines are in green. (B) Model of the bound state for the protein-DNA complex between *S. pneumoniae* ParC and the DNA E site [15]. The DNA is in Licorice mode and the protein is in cartoon mode. The positions on the DNA helix are given by numbers in yellow circles. The nucleotides are indicated by square boxes. Amino acids of the 100-122 loop are indicated by ovals with corresponding names. Helix α4 is in cyan and helix α3 is in yellow. Active site tyrosine and arginine are in CPK mode and the point of the DNA cleavage is indicated by yellow asterisk in red circle. Ser 79 and Asp 83 are shown using VDW representation and are in yellow and red respectively. The panel was generated using VMD [50] and Pov-Ray.
(3.14 MB PDF)

Movie S1 A schematic process of DNA binding and recognition by *S. pneumoniae* ParC. The process is shown both in cartoon and electrostatic potential surface modes. The positions of the active-site tyrosines are indicated by arrows when the tyrosines approach the target DNA backbone phosphates. The DNA backbone is in rose, the base pairs involved in sequence specific DNA recognition are in green, the nucleotides adjacent to the cleavage points are in purple in Licorice mode and the non-specific base pairs are in silver. The active-site tyrosines of the ParC dimer are represented in CPK mode in blue. The helix α3 is in cyan, the helix α4 is in red, the 100-122 loop is in yellow and the Gly 166-Pro 179 region is in orange. The movie was generated using VMD [50], Pov-Ray and PyMOL [53].
(4.77 MB MPG)
