# Supplementary figures and images for: Breakage-Reunion Domain of Streptococcus pneumoniae Topoisomerase IV: Crystal Structure of a Gram-Positive Quinolone Target
Source: PLoS One. 2007 Mar 21;2(3):e301. doi: 10.1371/journal.pone.0000301 (PMC1810434; doi:10.1371/journal.pone.0000301)

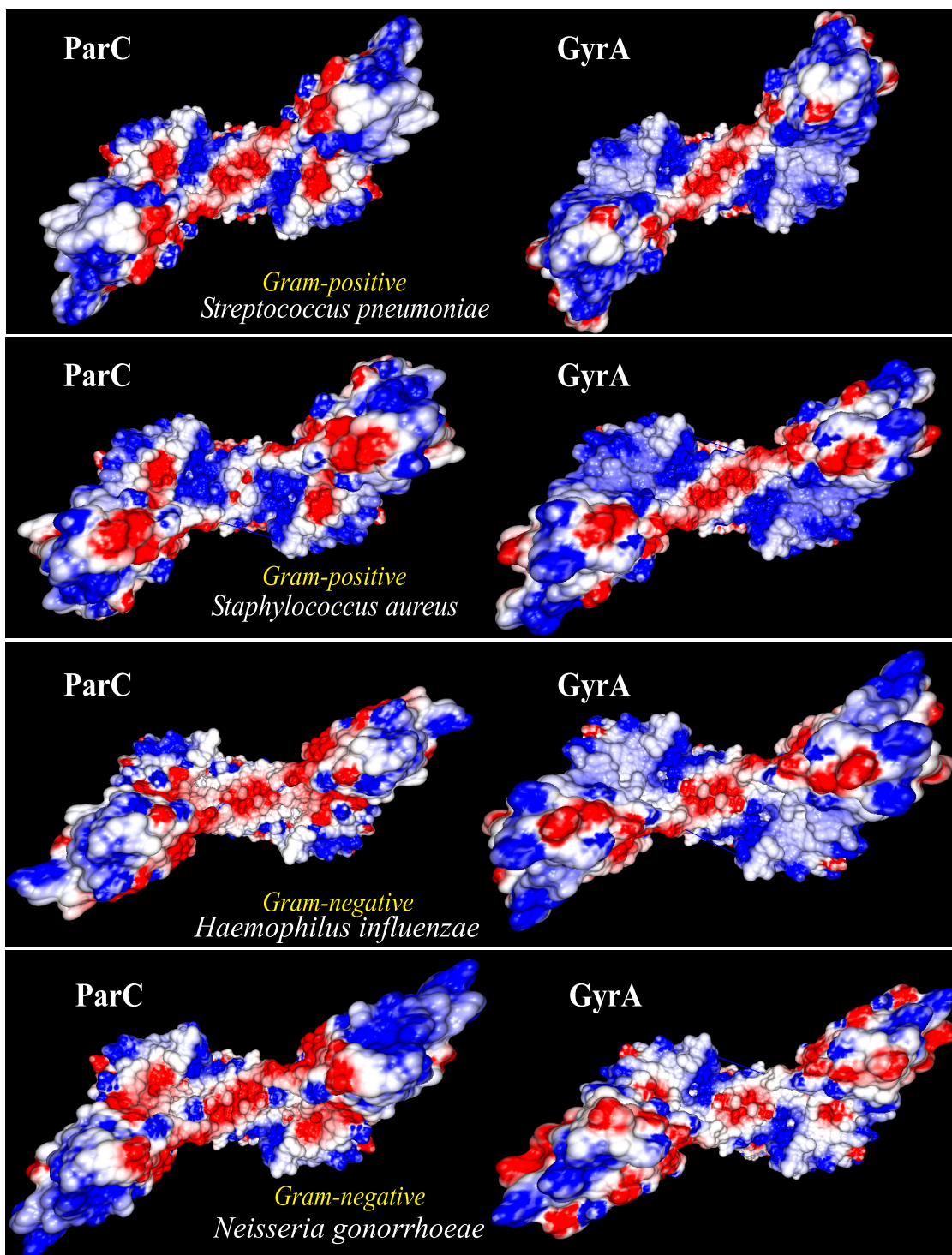

Supplement: Figure S1 — Electrostatic surface potential modeling. GRASP2 [51], [52] electrostatic surface potentials within the DNA-binding grooves calculated for DNA-binding domains of topoisomerases IIA from different organisms using models generated by 3D-JIGSAW [41]–[43] on the base of the known structure of E. coli GyrA (1AB4). (3.64 MB PDF) [file pone.0000301.s001.pdf]
