# Supplementary material for: Breakage-Reunion Domain of Streptococcus pneumoniae Topoisomerase IV: Crystal Structure of a Gram-Positive Quinolone Target
Source: PLoS One. 2007 Mar 21;2(3):e301. doi: 10.1371/journal.pone.0000301 (PMC1810434; doi:10.1371/journal.pone.0000301)

A

```
                          **   *  **       **  ***  **
GyrA E.coli        61 KAYKKSARVVGDVIGKYHPHGDSAVYDTIVRMAQPFSLRYMLVDGQGNFGSIDG-DSAAAMRYTE 124
GyrA S.pneumoniae  59 KPHKKSARITGDVMGKYHPHGDSSIYEAMVRMAQWWSYRYMLVDGHGNFGSMDG-DSAAAQRYTE 122
ParC S.pneumoniae  57 KSYRKSAKSVGNIMGNFHPHGDSSIYDAMVRMSQNWKNREILVEMHGNNGSMDG-DPPAAMRYTE 120
ParC E.coli        58 AKFKKSARTVGDVLGKYHPHGDSACYEAMVLMAQPFSYRYPLVDGQGNWGAPDDPKSFAAMRYTE 122
                          ------α3-----   ------α4------            -----100-122 loop--
```

B

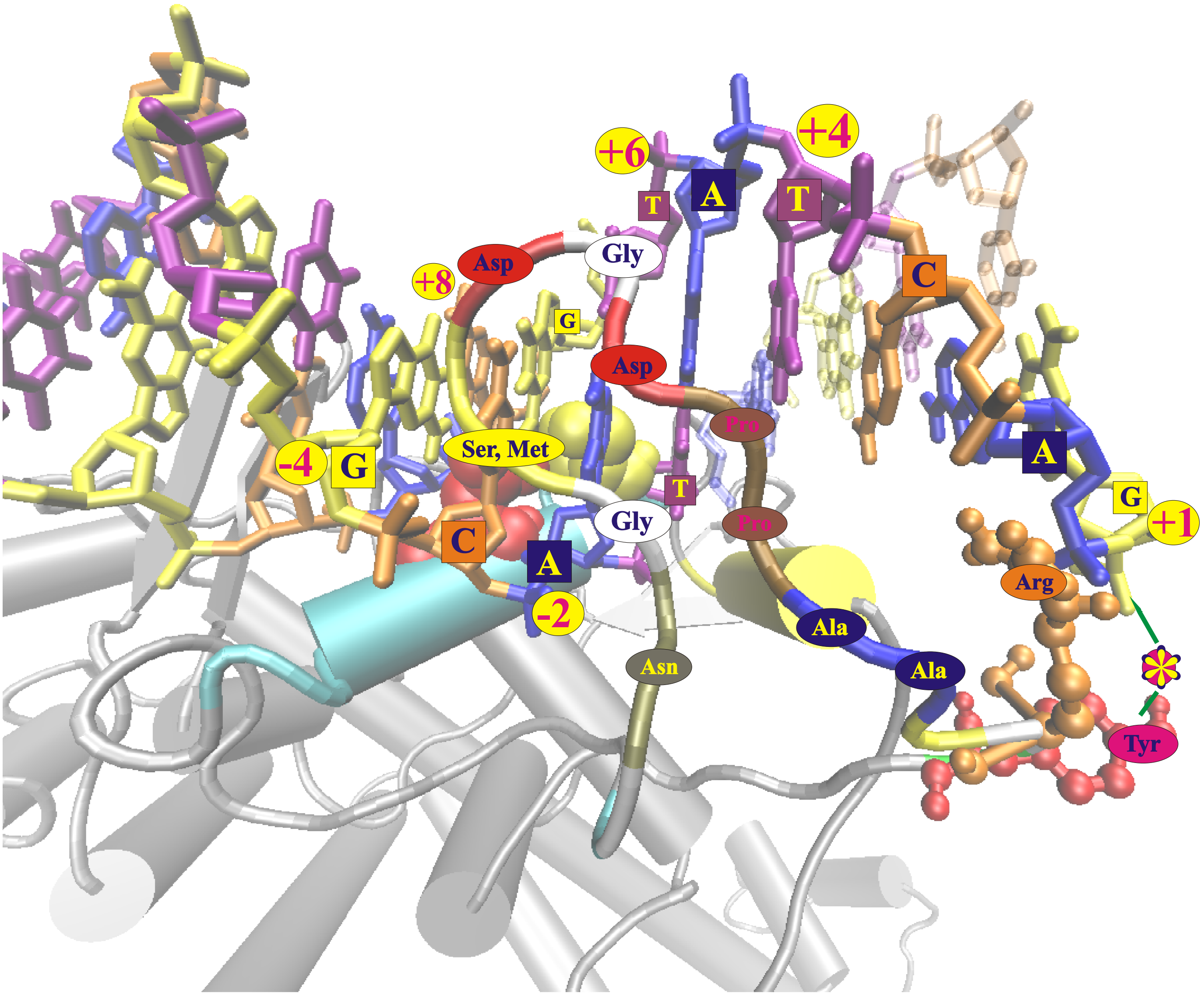

Supplement: Figure S2 — Modeling of the DNA sequence recognition by topoisomerase IV. (A) Amino acid sequence alignment for the regions of GyrA and ParC from E. coli and S. pneumoniae comprising helices α3 and α4 and the 100-122 loop. The residues within the α-helices which are likely to interact directly with the incoming DNA helix are indicated by color (red for α3 and blue for α4) and asterisks. Active site tyrosines and arginines are in green. (B) Model of the bound state for the protein-DNA complex between S. pneumoniae ParC and the DNA E site [15]. The DNA is in Licorice mode and the protein is in cartoon mode. The positions on the DNA helix are given by numbers in yellow circles. The nucleotides are indicated by square boxes. Amino acids of the 100-122 loop are indicated by ovals with corresponding names. Helix α4 is in cyan and helix α3 is in yellow. Active site tyrosine and arginine are in CPK mode and the point of the DNA cleavage is indicated by yellow asterisk in red circle. Ser 79 and Asp 83 are shown using VDW representation and are in yellow and red respectively. The panel was generated using VMD [50] and Pov-Ray. (3.14 MB PDF) [file pone.0000301.s002.pdf]
